# Overexpression of the dystrophins Dp40 and Dp40_L170P_ modifies neurite outgrowth and the protein expression profile of PC12 cells

**DOI:** 10.1038/s41598-022-05271-2

**Published:** 2022-01-26

**Authors:** César García-Cruz, Candelaria Merino-Jiménez, Jorge Aragón, Víctor Ceja, Brenda González-Assad, Juan Pablo Reyes-Grajeda, Cecilia Montanez

**Affiliations:** 1 Departamento de Genética y Biología Molecular, Centro de Investigación y de Estudios Avanzados del IPN, Mexico City, Mexico; 2 Instituto Nacional de Medicina Genómica, Mexico City, Mexico

**Keywords:** Genetics, Molecular biology, Neuroscience

## Abstract

Dp40 is ubiquitously expressed including the central nervous system. In addition to being present in the nucleus, membrane, and cytoplasm, Dp40 is detected in neurites and postsynaptic spines in hippocampal neurons. Although Dp40 is expressed from the same promoter as Dp71, its role in the cognitive impairment present in Duchenne muscular dystrophy patients is still unknown. Here, we studied the effects of overexpression of Dp40 and Dp40_L170P_ during the neuronal differentiation of PC12 Tet-On cells. We found that Dp40 overexpression increased the percentage of PC12 cells with neurites and neurite length, while Dp40_L170P_ overexpression decreased them compared to Dp40 overexpression. Two-dimensional gel electrophoresis analysis showed that the protein expression profile was modified in nerve growth factor-differentiated PC12-Dp40_L170P_ cells compared to that of the control cells (PC12 Tet-On). The proteins α-internexin and S100a6, involved in cytoskeletal structure, were upregulated. The expression of vesicle-associated membrane proteins increased in differentiated PC12-Dp40 cells, in contrast to PC12-Dp40_L170P_ cells, while neurofilament light-chain was decreased in both differentiated cells. These results suggest that Dp40 has an important role in the neuronal differentiation of PC12 cells through the regulation of proteins involved in neurofilaments and exocytosis of synaptic vesicles, functions that might be affected in PC12-Dp40_L170P_.

## Introduction

Dp40 is the smallest dystrophin reported to date and is transcribed from intron 62 to exon 70 of the *dmd* gene^1^. Moreover, the Dp40 transcript is expressed in several human foetal tissues, such as muscle, lung, liver, and brain, embryonic stem cells, adult muscle and the schwannoma cell line^1^. In addition, its expression has been reported in different regions of the brain, such as the cortex, cerebellum and hippocampus of mice^1,2^. Dp40 mRNA is expressed in undifferentiated and nerve growth factor (NGF) differentiated PC12 cells^3^. Unlike other Dp71 isoforms, Dp40 lacks the C-terminal end and therefore the motifs and domains that interact with syntrophins and dystrobrevins^4^. Similar to Dp71, Dp40 contains part of the WW domain, which provides the main binding site to β-dystroglycan (β-DG), a component of the dystrophin-associated protein complex (DAPC), as well as the EF-hand motifs and the ZZ domain involved in Ca^2+^ binding and transport to the nucleus, respectively^4–6^. The EF-hand motifs and ZZ domain are necessary for the WW domain to interact efficiently with β-DG^7,8^. It has been reported that Dp40 interacts with syntaxin 1A (STX1A), vesicle-associated membrane protein 2 (VAMP2) and synaptosome-associated protein 25 (SNAP25), a group of presynaptic proteins involved in exocytosis of synaptic vesicles of the hippocampus and cortex in the mouse brain^9^. In addition, Dp40 protein shows high expression in neuronal cells and a decrease in non-neuronal cells in primary culture of the mouse hippocampus. Moreover, the Dp40 protein is only expressed in postnatal stages, not in the embryonic stages of the mouse brain^2^. In PC12 cells, transient expression of Dp40 showed that it is located in the membrane and cytoplasm of undifferentiated cells, while its subcellular distribution changes in NGF-differentiated PC12 cells at day 3 post-treatment, which it is located in the membrane and cytoplasm with a significant increase in the nucleus. In addition, the mutant Dp40_L170P_ has a change in residue 170 from leucine to proline, promoting exclusive nuclear localization in PC12 cells^3^. Furthermore, Dp40 colocalized with β-DG opposite to Dp40_L170P_ in NGF-differentiated PC12 cells^3^. Additionally, primary culture of mouse brain neurons showed that Dp40 is located in the membrane, nucleus and excitatory dendritic spines^2^.

One-third of patients with Duchenne muscular dystrophy (DMD) have different degrees of cognitive deficits coupled with progressive muscular degeneration that characterize the disease. Cognitive impairment in DMD patients has been mostly associated with alterations in dystrophin Dp71 expression^10^, and the disruption of Dp71 was shown to alter DAPC^11^. Although Dp40 is ubiquitously expressed from the same promoter as Dp71^1^, its participation in DAPC and therefore in this disease is still unknown. Interestingly, a report showed that six patients with different degrees of cognitive deficit had a deletion of three base pairs at positions 9711–9714 in the *dmd* gene^12^. Importantly, this deletion is located in the same residue where the punctual change from leucine to proline is located in the mutant of Dp40 (Dp40_L170P_). Therefore, in this work, to contribute to the knowledge of Dp40 function, we created PC12 Tet-On cells that overexpress dystrophin Dp40 or Dp40_L170P_ in an inducible and stable manner to analyze the effect of overexpression of these proteins on the neurite outgrowth process through morphometric and proteomic analyses. The results obtained showed that Dp40 overexpression stimulates neurite outgrowth in the opposite manner as Dp40_L170P_, which its expression caused a reduction in neurite number and length. In addition, we carried out a proteomic analysis using two-dimensional gel electrophoresis (2-DE) to compare the Dp40_L170P_ expression profile with that of the control. We identified proteins related to alteration of the neurite outgrowth process. Dp40_L170P_ increased the expression levels of α-internexin and S100a6, proteins involved in intermediate filaments and reorganization of the cytoskeleton, respectively. Additionally, we evaluated the expression of VAMP, NF-L and HspB1, which are related to secretory processes, neurofilaments and cytoskeletal remodelling. The results of this study provide valuable information about the role of Dp40 in neurite outgrowth during neural differentiation and its participation in cognitive deficits when the Dp40 isoform is disrupted.

## Results

### Overexpression of Myc-Dp40 and Myc-Dp40_L170P_ showed different effects on the neurite outgrowth of PC12-Tet-On cells

**Figure 1:**
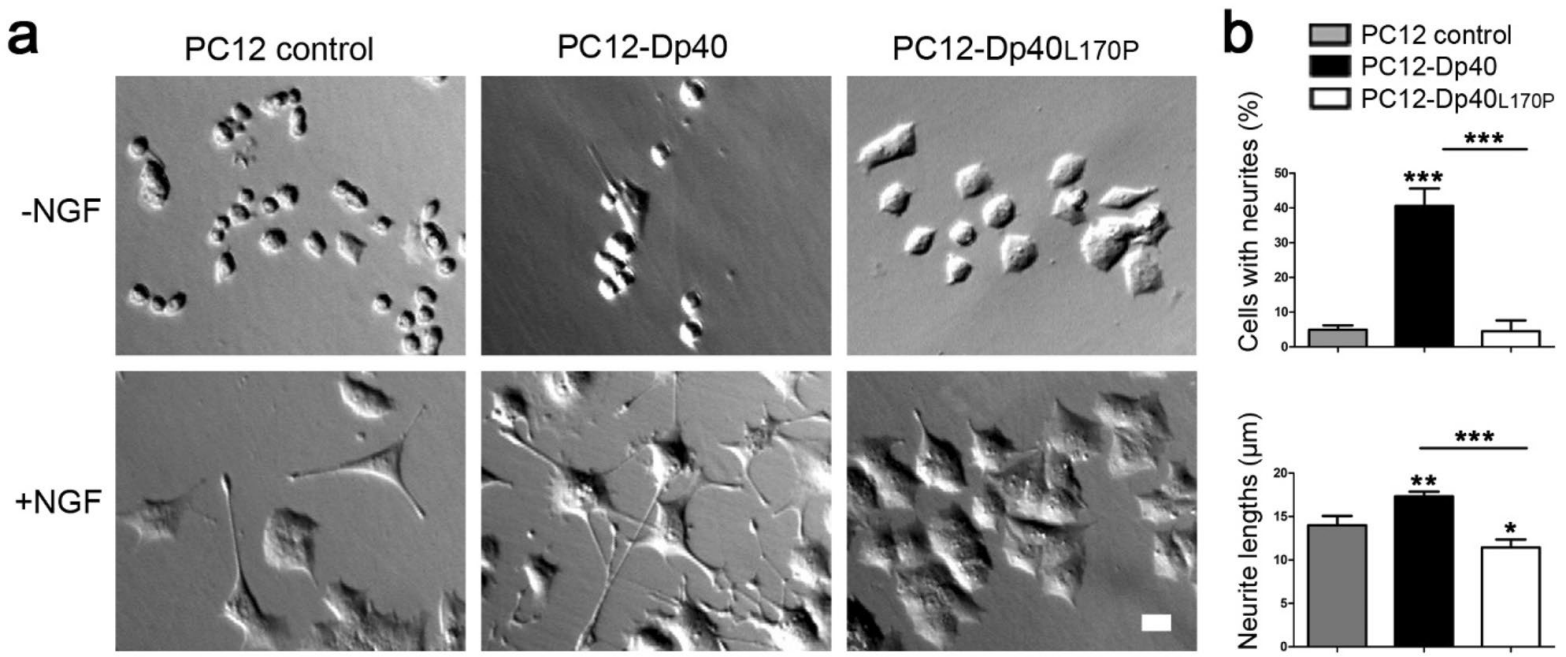
Morphometric analysis of undifferentiated and NGF-differentiated PC12-Dp40 and PC12-Dp40_L170P_ cells. PC12 control, PC12-Dp40 and PC12-Dp40_L170P_ cells were differentiated with NGF for 9 days. (**a**) Morphometric analysis was performed using clear-field micrographs of the undifferentiated (top panels) and differentiated PC12 cells (bottom panels). (**b**) Quantification of the differentiation ratio of the NGF-differentiated PC12 control, PC12-Dp40 and PC12-Dp40_L170P_ cells. Top panel*:* percentage of cells with neurites. Bottom panel*:* average of neurite lengths. *P ˂ 0.05, **P ˂ 0.01, ***P ˂ 0.0005 compared to the PC12 control or PC12-Dp40 versus PC12-Dp40_L170P_ cells. The scale bar represents 10 µm.

PC12 Tet-On cells were stably transfected with the pTRE2pur-Myc/Dp40 or pTRE2pur-Myc/Dp40_L170P_ vector, and vector integration was tested by genomic DNA PCR (Supplementary Figure S1). To characterize the isolated clones PC12-Dp40 and PC12-Dp40_L170P_, we determined the minimum concentration of doxycycline to induce the overexpression of the recombinant proteins through western blotting (WB) using an anti-c-Myc antibody. Myc-Dp40 and Myc-Dp40_L170P_ were overexpressed using 50–1000 ng/ml doxycycline in undifferentiated PC12 Tet-On cells (Supplementary Figure S2). Because higher concentrations of doxycycline did not result in an increase in recombinant protein expression, we used 100 ng/ml doxycycline to overexpress Dp40 proteins to eliminate the cytotoxic effect of doxycycline reported at 200 ng/ml^13^. With this doxycycline concentration, undifferentiated and NGF-differentiated PC12 Tet-On cells were analyzed to evaluate the morphological effect of Myc-Dp40 and Myc-Dp40_L170P_ overexpression on neurite outgrowth processes compared with those of the PC12 control cells. Myc-Dp40 and Myc-Dp40_L170P_ overexpression did not affect the morphology of the undifferentiated PC12 cells (Fig. 1a, top panels), in contrast to the NGF-differentiated PC12 cells (Fig. 1a, bottom panels). The differentiation ratio (Fig. 1b, top panel) showed that the PC12-Dp40 cells had a greater number of cells with neurites than the PC12 control cells (40.6% ± 2.90 versus 4.96% ± 0.72; *P* = 0.0003), while PC12-Dp40_L170P_ did not result in a significant difference compared with the PC12 control cells. However, a decrease in the differentiation ratio of the NGF-differentiated Dp40_L170P_ cells was observed compared with that of the PC12-Dp40 cells (4.50% ± 1.82 versus 40.6% ± 2.90; *P* = 0.0005). Additionally, neurite length quantification (Fig. 1b, bottom panel) showed that the NGF-differentiated PC12-Dp40 cells had longer neurites than the PC12 control cells (17.3 µm ± 0.30 versus 14.0 µm ± 0.62; *P* = 0.0086). However, the PC12 control cells presented longer neurites than the PC12-Dp40_L170P_ cells (14.0 µm ± 0.62 versus 11.5 µm ± 0.052; *P* = 0.0359). Therefore, the differentiated PC12-Dp40_L170P_ cells showed lower neurite outgrowth than the PC12-Dp40 cells (11.5 µm ± 0.52 versus 17.3 µm ± 0.30; *P* = 0.006). Based on these results, Myc-Dp40 expression stimulates neurite outgrowth, whereas Myc-Dp40_L170P_ inhibits this process.

### Protein expression profile of differentiated PC12-Dp40_L170P_ cells

**Figure 2:**
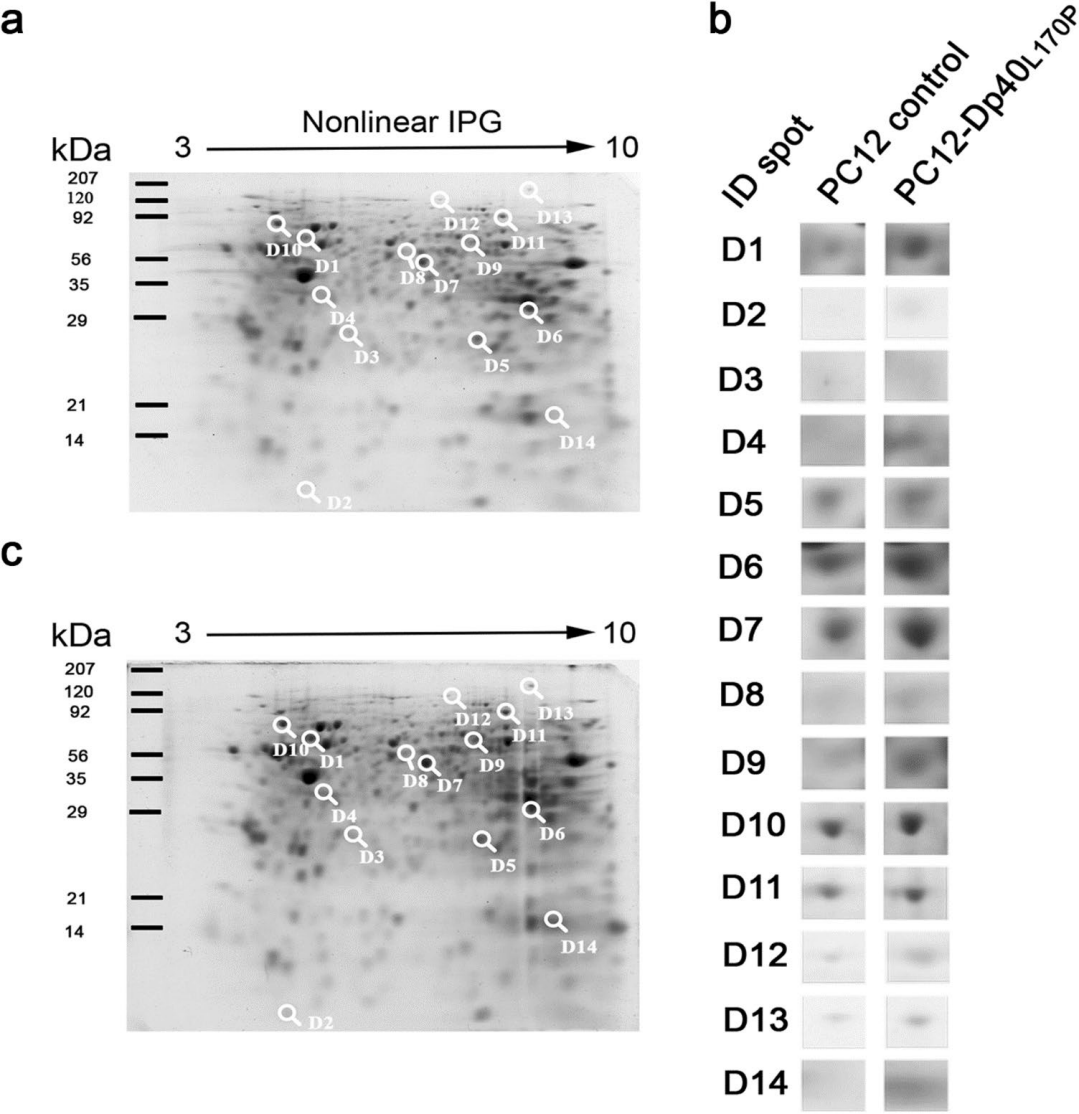
Two-dimensional gel electrophoresis images of proteins from the NGF-differentiated PC12 control and PC12-Dp40_L170P_ cells. Total protein extracts of the NGF-differentiated PC12 control and PC12-Dp40_L170P_ cells from three independent experiments were collected after differentiation for 9 days and separated by isoelectric focusing using immobilized pH nonlinear strips (7 cm), followed by SDS-PAGE. Gels were stained with coomassie colloidal blue. (**a**) Protein profile of the PC12 control cells and (**b**) the PC12-Dp40_L170P_ cells. Numbers 1–14 indicate the IDs of the differentially expressed protein spots. (**c**) Magnified views of the differentially expressed protein spots. Arrows represent the nonlinear immobilized pH gradient used for IEF. The positions of standard markers for the second dimension are shown on the left side of each image.

To understand the inhibitory mechanism of neurite outgrowth produced by Dp40_L170P_, we analyzed the total protein extracts of the PC12 control and PC12-Dp40_L170P_ NGF-differentiated cells for 9 days by 2-DE (Fig. 2a,b). Differentially expressed protein spots with at least a 1.1-fold change were considered differentially expressed. Among the 344 protein spots detected, 14 (spots ID: 1–14) showed differential expression (Fig. 2c), 13 proteins showed upregulated expression and one showed downregulated expression, and magnified views of the differentially expressed protein spots are presented in Fig. 2c. Thus, Dp40_L170P_ modified the protein expression profile of PC12 Tet-On cells. The 14 spots selected were excised from the 2-DE gels and analyzed by MS. The details of each identified protein are summarized in Table 1. The proteins with highly upregulated expression were S100a6 (2.1-fold, p*I* 5.2) and α-internexin (2.0-fold, p*I* 5.3), which are involved in the reorganization of cytoskeletal structure and neurofilaments present in immature neurons, respectively. Only the expression of ectonucleotide pyrophosphatase (1.5-fold, p*I* 5.5), a protein related to cellular communication, was downregulated. The other proteins identified were related to chaperone-like activity (T-complex protein 1 subunit zeta and the endoplasmic reticulum chaperone BIP) and metabolism (phosphoglycerate mutase 1, l-lactate dehydrogenase A chain and alpha enolase). This last group of proteins usually shows upregulated expression in several proteomic analyses, probably due to cellular stress responses^14^.

**Table 1:** Proteins identified by mass spectrometry.

Spot ID	Variation	Fold change in spot	MS	Accession no.^a^	Protein	Gene	MW (kDa)	pI	Match pept	SC (%)^b^
1	+	2	MALDI	P23565	Alpha internexin	Ina	56.11	5.20	14	29.8
2	+	2.1	MALDI	P05964	S100A6 isoform	S100a6	10.03	5.30	1	58.4
5	+	1.15	MALDI	P25113	Phosphoglycerate mutase 1	Pgam1	28.83	6.67	4	29.1
6	+	1.47	MALDI	P04642	l-Lactate dehydrogenase A chain	Ldha	36.45	8.45	2	21.4
7	+	1.41	MALDI	P04764	Alpha enolase	Eno1	47.12	6.12	12	45.3
8	−	1.52	MALDI	P84039	Ectonucleotide pyrophosfatase/phosphodiesterase	Enpp5	54.38	5.52	1	16.4
9	+	1.56	MALDI	Q3MHS9	T-complex protein 1 subunit zeta	Cct6a	58.01	6.46	2	10.5
10	+	1.20	MALDI	P06761	Endoplasmic reticulum chaperone BIP	Hspa5	72.33	5.07	5	17.6

^a^Accession number according to the swiss-prot *Rattus novergicus* database.

^b^SC % = % of the sequence identified.

### Myc-Dp40 and Myc-Dp40_L170P_ overexpression modified the expression of α-internexin, VAMP, HspB1 and NF-L during the neuronal differentiation process

**Figure 3:**
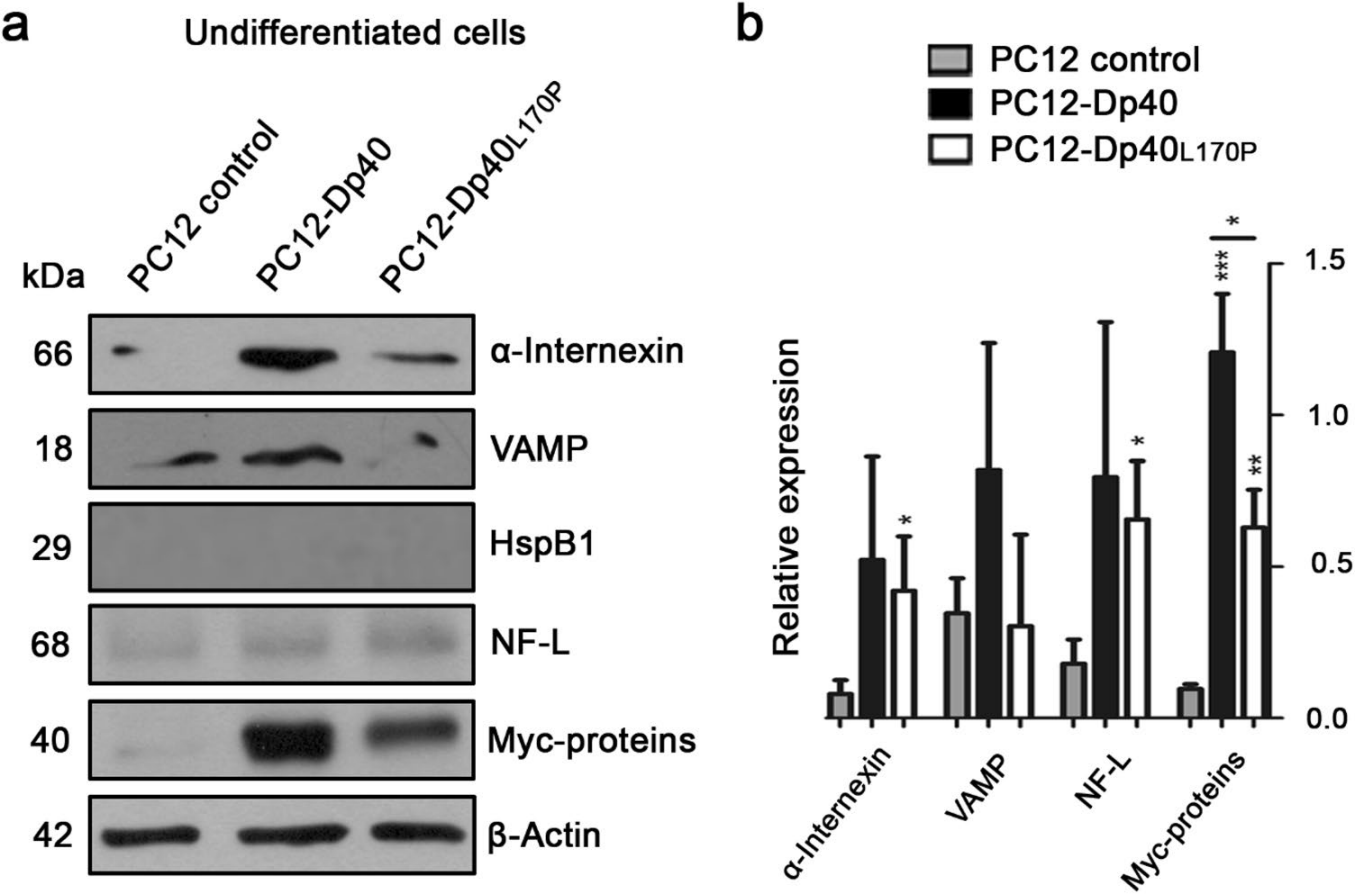
Expression of the proteins α-internexin, VAMP, HspB1, NF-L, Myc-Dp40 and Myc-Dp40_L170P_ in the undifferentiated PC12-Dp40 and PC12-Dp40_L170P_ cells. Protein extracts were obtained from the undifferentiated PC12 control, PC12-Dp40 and Dp40_L170P_ cells and analyzed by WB. (**a**) Expression of α-internexin, VAMP, HspB1, NF-L, HspB1, Myc-Dp40 and Myc-Dp40_L170P_. (**b**) Relative expression of α-internexin, VAMP, and NF-L was determined as indicated in the materials and methods. The graph represents the mean ± SD from three independent experiments. *P ˂ 0.034 and 0.016 for α-internexin and NF-L comparing the PC12 control cells and the PC12-Dp40_L170P_ cells. ***P ˂ 0.0006 and **P ˂ 0.0018 for Myc-Dp40 and Myc-Dp40_L170P_ comparing the PC12-Dp40 and PC12-Dp40_L170P_ with PC12 control cells. *P ˂ 0.0124 for Myc-Dp40 comparing the PC12-Dp40 and PC12-Dp40_L170P_ cells. β-Actin was used as a loading control. Molecular weight of each protein is indicated in kDa. The blots of each protein are available in Supplementary Fig. 3.

**Figure 4:**
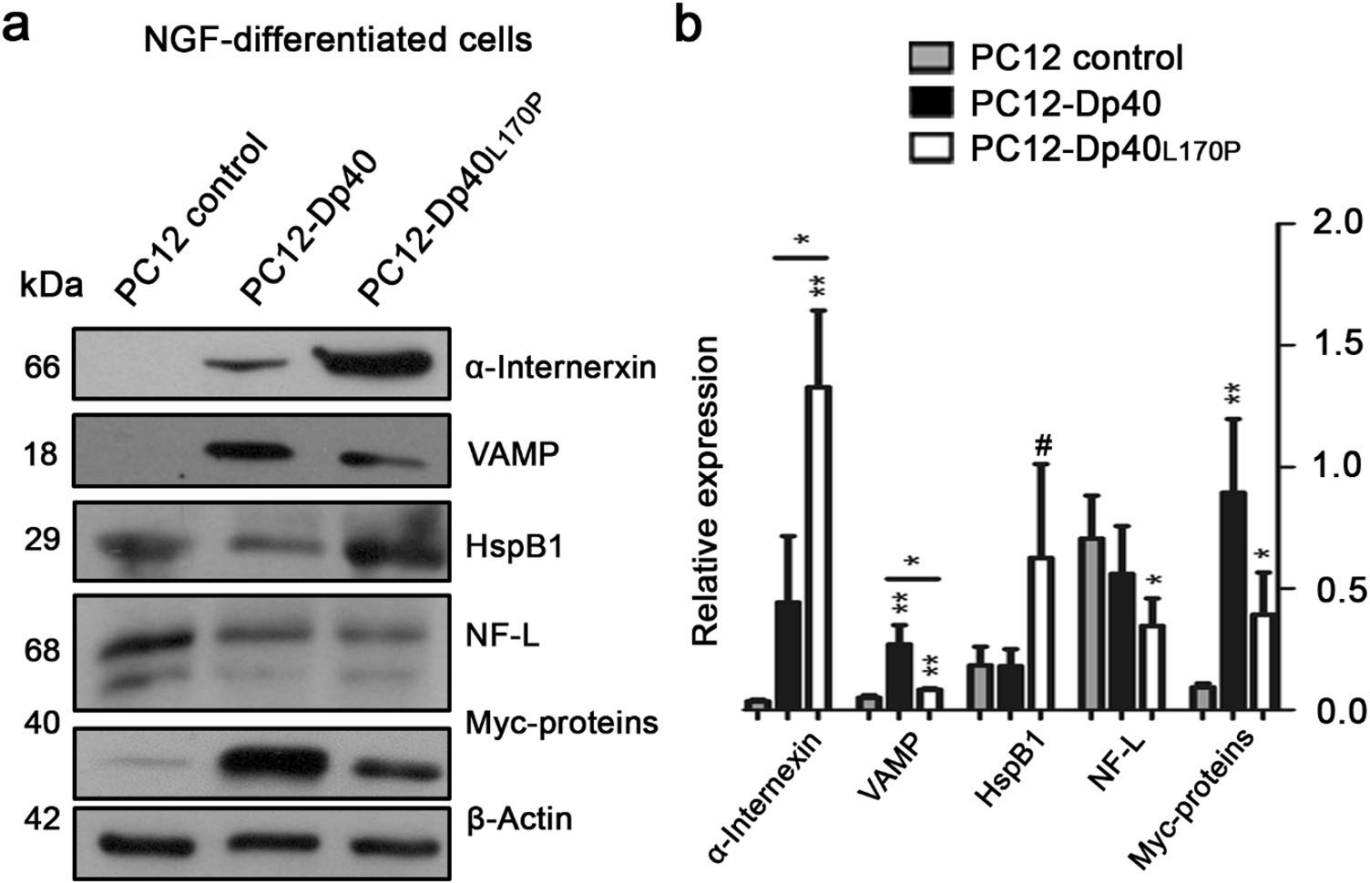
Differential expression of α-internexin, VAMP, HspB1, NF-L, Myc-Dp40 and Myc-Dp40_L170P_ in the NGF-differentiated PC12-Dp40 and PC12-Dp40_L170P_ cells. Protein extracts were obtained from the NGF-differentiated PC12 control, PC12-Dp40 and PC12-Dp40_L170P_ cells and analyzed by WB. (**a**) Expression of α-internexin, VAMP, HspB1, NF-L, Myc-Dp40 and Myc-Dp40_L170P_. (**b**) Relative expression of α-internexin, VAMP, HspB1, NF-L, Myc-Dp40 and Myc-Dp40_L170P_ was determined as indicated in the materials and methods. The graph presents the mean ± SD from three independent experiments. **P ˂ 0.002 and *P ˂ 0.021 for α-internexin comparing the PC12-Dp40_L170P_ with PC12 control and PC12-Dp40 cells, respectively. **P ˂ 0.008 and **P ˂ 0.007 for VAMP comparing the PC12 control with PC12-Dp40 and PC12-Dp40_L170P_ cells, respectively, and *P ˂ 0.015 comparing PC12-Dp40 and PC12-Dp40_L170P_. *P ˂ 0.040 for NF-L comparing the PC12 control and PC12-Dp40_L170P_ cells. *P ˂ 0.010 and *P ˂ 0.040 for Myc-Dp40 and Myc-Dp40_L170P_ comparing the PC12-Dp40 and PC12-Dp40_L170P_ with PC12 control cells. No statistical difference was observed for HspB1 (^#^*P* > 0.1). β-Actin was used as a loading control. Molecular weight of each protein is indicated in kDa. The blots of each protein are available in Supplementary Fig. 4.

After MS identification, we validated the differential expression of α-internexin, one of the top two proteins with upregulated expression determined by WB in undifferentiated (Fig. 3) and differentiated (Fig. 4) PC12 control, PC12-Dp40 and PC12-Dp40_L170P_ cells. First, we evaluated the expression of the recombinant proteins: the Myc-Dp40 and Myc-Dp40_L170P_ proteins were overexpressed 13.3- and 7-fold in the undifferentiated PC12-Dp40 and PC12-Dp40_L170P_ cells, respectively, showing a significant increase compared to those of the PC12 control cells. In addition, Myc-Dp40 (1.9-fold) was more highly expressed than Myc-Dp40_L170P_ in the PC12-Dp40 and PC12-Dp40_L170P_ cells. Alpha-internexin, which is involved in the expression and assembly of neurofilaments in the central nervous system^15,16^, presented a 5.25-fold increase in the PC12-Dp40_L170P_ cells compared with the PC12 control cells. However, α-internexin expression was not significantly different in the undifferentiated PC12-Dp40 cells compared with the PC12 control and PC12-Dp40_L170P_ cells. Since a previous report^9^ showed that VAMP interacts with the Dp40 protein, we decided to validate its expression. The results showed that VAMP is expressed at low levels and without a significant difference in all undifferentiated cells. Recently, it was observed that Dp71e_Δ71_ and mutant Dp71_Δ78-79_ overexpression increased the HspB1, a remodeler of the cytoskeleton, during the neuronal differentiation process of PC12 cells^17,18^. Thus, we analyzed the expression of the HspB1 protein; however, we did not detect this protein under undifferentiated conditions. In addition, NF-L expression was evaluated because it is a neuronal differentiation marker that is expressed in postmitotic neurons as part of the cytoskeletal structure. However, we did not observe a significant difference in NF-L expression in any undifferentiated PC12 cells.

In the NGF-differentiated PC12 cells (Fig. 4), Myc-Dp40 and Myc-Dp40_L170P_ expression showed an increase of 9.8- and 4.3-fold in the PC12-Dp40 and PC12-Dp40_L170P_ cells, respectively compared with the PC12 control cells. However, there was no significant difference between Myc-Dp40 and Myc-Dp40_L170P_. Alpha-internexin expression in the PC12-Dp40 cells did not show a significant difference compared with that in the PC12 control cells. However, its expression in the PC12-Dp40_L170P_ cells was increased 44- and 3-fold compared with that in the PC12 control and PC12-Dp40 cells, respectively. This increase is in accordance with what we observed in the 2-DE gels in the differentiated PC12 control cells and the PC12-Dp40_L170P_ cells. VAMP expression was increased 5.4- and 1.6-fold in the NGF-differentiated PC12-Dp40 and PC12-Dp40_L170P_ cells, respectively, compared with that in the PC12 control cells. However, the PC12-Dp40 cells showed an expression increase of 3.3-fold compared to the PC12-Dp40_L170P_ cells. HspB1 presented low expression and no significant difference in the differentiated cell lines was observed (Fig. 4b). NF-L expression was not significantly different between the differentiated PC12 control and PC12-Dp40 cells. In contrast, the PC12 control cells showed an increase of 2-fold compared with the PC12-Dp40_L170P_ cells but no significant difference compared with the PC12-Dp40 cells. All these results showed that the disruption of dystrophin Dp40 through the expression of the mutant Dp40_L170P_ could affect the expression of neurofilaments such as α-internexin and NF-L during the neuronal differentiation process of PC12 cells.

### Differential distribution of the Myc-Dp40, Myc-Dp40_L170P_ and α-internexin proteins in PC12-Tet-On cells

**Figure 5:**
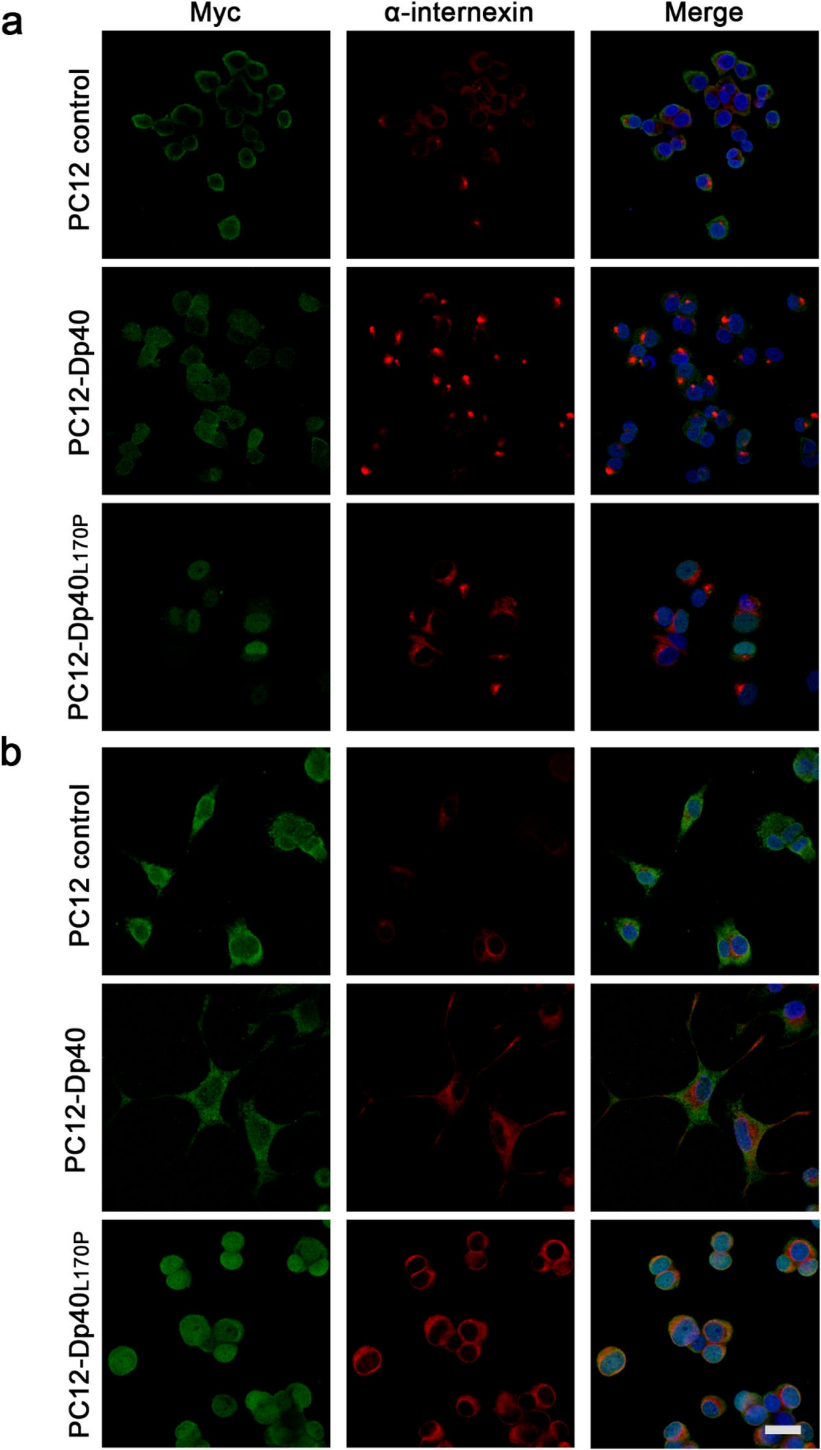
Cellular distribution of α-internexin in undifferentiated and NGF-differentiated PC12-Dp40 and PC12-Dp40_L170P_ cells. ImmF was performed on the PC12-Dp40, PC12-Dp40_L170P_ and PC12 control cells after the induction of differentiation. ImmF staining for the Myc-Dp40 and Myc-Dp40_L170P_ proteins was performed using the anti-c-Myc antibody (green) and α-internexin (red). Nuclei were stained with DAPI (blue). Images represent a single equatorial Z-section from confocal images to show the localization of each protein. (**a**) Undifferentiated and (**b**) differentiated PC12 control, PC12-Dp40 and PC12-Dp40_L170P_ cells. Merged images correspond to the overlap of Myc-Dp40 and Myc-Dp40_L170P_ with the α-internexin protein. The figure is a representative result of three independent experiments. The scale bar represents 20 µm.

Differences in the subcellular distribution of Myc-Dp40, Myc-Dp40_L170P_ and c-Myc epitopes were evaluated through indirect immunofluorescence (ImmF) assays in the undifferentiated and NGF-differentiated PC12 Tet-On cells (Fig. 5a,b, respectively). Myc-Dp40 protein localization did not present a significant difference between the cytoplasm and nucleus (49.08% ± 2.20 versus 50.92% ± 2.20; *P* = 0.55) in the undifferentiated cells, while Myc-Dp40_L170P_ showed an increase in the nucleus compared to the cytoplasm (75.06% ± 1.6 versus 24.94% ± 1.69; *P* < 0.0001), unlike the c-Myc peptide in the PC12 control cells, which presented an increase in immunoreactivity in the cytoplasm compared to the nucleus (66.53% ± 1.58 versus 33.47% ± 1.580; *P* < 0.0001). Under NGF-differentiated conditions, in the PC12 control cells, the c-Myc-peptide was localized to the same extent in the cytoplasm and nucleus (52.68% ± 2.98 versus 47.32% ± 2.989; *P* = 0.21). However, Myc-Dp40 was mainly located in the cytoplasm compared to the nucleus (61.58% ± 3.00 versus 38.42% ± 3.00; *P* < 0.0001), while Myc-Dp40_L170P_ showed higher immunoreactivity in the nucleus than in the cytoplasm (62.20% ± 2.19 versus 37.80% ± 2.19; *P* < 0.0001).

Alpha-internexin has been detected in the cytoplasm of postmitotic neurons of the periphery and central nervous system^19,20^. In the undifferentiated PC12 control, PC12-Dp40 and PC12-Dp40_L170P_ cells, α-internexin was observed in the cytoplasm. We found that the immunoreactivity of this protein was similar in the undifferentiated PC12 control (0.73% ± 0.19), PC12-Dp40 (0.83% ± 0.08; *P* = 0.67) and PC12-Dp40_L170P_ cells (1.42% ± 0.37; *P* = 0.18). However, the NGF-differentiated PC12-Dp40_L170P_ cells (3.03% ± 0.10) showed a higher percentage of immunoreactivity than the PC12-Dp40 (1.76% ± 0.28; *P* = 0.013) and PC12 control cells (1.52% ± 0.21; *P* = 0.003), but we did not observe a significant difference between the NGF-differentiated PC12-Dp40 (0.44% ± 0.157; *P* = 0.53) and PC12 control cells (0.36% ± 0.003). In the NGF-differentiated PC12-Dp40 cells, α-internexin was observed in the cytoplasm and along the length of neurites, while in the PC12 control and PC12-Dp40_L170P_ cells, it was mostly distributed in the cytoplasm. Based on these results, the subcellular distributions of Myc-Dp40 and Myc-Dp40_L170P_ were mostly cytoplasmic and nuclear, respectively. In addition, the overexpression of Myc-Dp40_L170P_ increases α-internexin expression in the undifferentiated and NGF-differentiated PC12-Dp40_L170P_ cells.

## Discussion

PC12 cells are a neuronal differentiation model widely used to study the reorganization of the cytoskeleton, electrical excitability and secretory vesicle dynamics during the neuronal differentiation process due to their similarities to sympathetic neurons^21–23^. Previous studies have shown that PC12 expresses short dystrophins, including Dp71 isoforms from Dp71d, Dp71f, Dp71e groups and Dp40^3,24,25^ and some of them show increased expression during the neuronal differentiation process^24^. Interestingly, through antisense technology against the 5﻿′ terminus of short dystrophin mRNAs (Dp71/Dp40), it has been shown that these dystrophins are essential for neurite outgrowth^26^. In addition, one study revealed that Dp40 accumulates in the nucleus during the neuronal differentiation process of PC12 cells^3^. Moreover, the exchange of leucine to proline in residue 170 of Dp40 (named Dp40_L170P_) promotes exclusive nuclear localization of Dp40, probably because it disrupts a nuclear export signal, which decreases the colocalization percentage between Dp40 and β-DG in PC12 cells^3,27^.

To obtain a better understanding of the role of dystrophin Dp40 in the neurite outgrowth process, we evaluated the effect of Dp40 and Dp40_L170P_ overexpression during neuronal differentiation of PC12 Tet-On cells. As has been reported for Dp71_Δ78-79_ and Dp71e_Δ71_^18,28^, Dp40 overexpression also stimulated neurite outgrowth by increasing the ratio of cells with neurites and the neurite length in PC12 cells. However, Dp40_L170P_ decreased the neurite length compared with those of the PC12-Dp40 and PC12 control cells (Fig. 1). This result suggests that Dp40 overexpression could stimulate the neurite outgrowth process through the subcellular distribution of Dp40. Moreover, Dp40 has been detected in neurites and dendritic spines in PC12 cells and hippocampal neurons in primary culture^2,3^. In addition, in this study, we observed that Dp40 was present in the neurites and cytoplasm and to a lesser extent in the nucleus of the NGF-differentiated PC12-Dp40 cells (Fig. 5). However, unlike what was previously reported^3^, where Dp40_L170P_ was located exclusively in the nucleus, in this work, Dp40_L170P_ not only increased its presence in the nucleus but was also observed in the cytoplasm of the undifferentiated and NGF-differentiated PC12-Dp40_L170P_ cells. We speculated that these differences in the subcellular distribution compared with the previous findings^3^ are because recombinant protein overexpression (Myc-Dp40 and Myc-Dp40_L170P_) was mediated through stable transfection in the Tet-On system and not through transient transfection in wild-type PC12 cells, as in the previous report.

The differentially expressed proteins identified through MS were related to the reorganization of the cytoskeleton (S100a6, α-internexin), cellular communication (ectonucleotide pyrophosphatase), chaperone-like activity (T-complex protein 1 subunit zeta and the endoplasmic reticulum chaperone BIP) and metabolism (phosphoglycerate mutase 1, L-lactate dehydrogenase A chain and α-enolase) according to the Protein Data Bank. Additionally, it has been reported that one-third of the differentially regulated proteins in proteomic studies are involved in metabolism, possibly because of the treatment to obtain the protein extract^14^. The protein with the highest differential expression was S100a6, with an increase of 2.1-fold (Table 1). The S100a6 protein belongs to the calcium binding protein family, which has been associated with several processes, such as proliferation, apoptosis cytoskeletal dynamics, and cellular response to stress factors^29^, including stimulating neurite outgrowth in PC12 cells^30^. However, the expression of this protein was upregulated in the PC12-Dp40_L170P_ cells, which showed lower neurite outgrowth than the PC12-Dp40 cells. Therefore, it is likely that S100a6 overexpression is not the only element required to promote neurite outgrowth.

Alpha-internexin was the second protein with the greatest upregulation in expression, with an increase of 2.0-fold. This protein belongs to intermediate filament type IV and is expressed early during brain development in most neurons^19^. After validation of the α-internexin expression, we observed that this protein showed decreased expression from undifferentiated to differentiated PC12 control cells (Figs. 3, 4), in which it was practically absent, consistent with the high α-internexin expression detected in immature myenteric neurons and its decrease with age^31^. However, α-internexin expression was highly expressed in the NGF-differentiated PC12-Dp40_L170P_ cells compared with the PC12 control and PC12-Dp40 cells. This finding contrasts with the fact that α-internexin decreases its expression in myenteric neurons during development^19^. Another study reported that α-internexin expression is upregulated after peripheral nerve injury in facial motor neurons of rats^32^, possibly as a compensatory mechanism to reassemble other neurofilaments and promote neurite outgrowth. Also, it was reported that α-internexin is the only neurofilament capable of coassembling itself^33^, with other neurofilaments increasing interfilament spacing^34^, probably to allow the assembly of other neurofilaments and promote neurite outgrowth. In addition, α-internexin has been identified as a candidate involved in early stages of brain regeneration in lesion models in the cerebellum^35^, a brain structure that has been suggested to participate in integrating learning processes^36^. However, the overexpression of α-internexin in transgenic mice induced abnormal swelling of Purkinje cell axons in the cerebellum, promoting neuronal dysfunction^37^, which could be related to the disruption of neurite outgrowth in the PC12-Dp40_L170P_ cells. Interestingly, the increase in α-internexin detected in the differentiated PC12-Dp40 and PC12-Dp40_L170P_ cells was not proportional to the amount of recombinant proteins produced (Figs. 3, 4) because Myc-Dp40 is more highly expressed than Myc-Dp40_L170P_ in the undifferentiated and differentiated PC12 cells. Therefore, differences in the expression levels of Myc-Dp40 and Myc-Dp40_L170P_ do not influence α-internexin expression. Thus, it is possible that the increase in α-internexin expression in the differentiated PC12-Dp40_L170P_ cells is due to the nuclear distribution of Myc-Dp40_L170P_ instead of protein production. Moreover, NF-L presented low expression in the PC12-Dp40_L170P_ cells, in contrast to the PC12 control cells, which showed high expression (Fig. 4). It has been reported that the later neuronal marker NF-L increases its expression during neuronal differentiation^38^; therefore, the low expression of NF-L in the differentiated PC12-Dp40_L170P_ suggests that the disruption of dystrophin Dp40 could alter the expression and function of neurofilaments affecting neurite outgrowth because the coordination between membrane trafficking and cytoskeletal remodelling are critical requirements for axonal growth^39^.

Dp40 overexpression stimulated the neurite outgrowth during neuronal differentiation, which could be related to the increase in VAMP in the differentiated PC12-Dp40 cells and its decrease in the PC12-Dp40_L170P_ cells (Fig. 4), where neurite outgrowth is disrupted. VAMP2 overexpression in PC12 cells stimulates neurite outgrowth^40^, and Dp40 interacts with VAMP2 in synaptic vesicle fractions in the adult mouse brain and colocalizes with VAMP in neurites of primary cultured hippocampal neurons^9^. Thus, we suggest that Dp40 overexpression promoted neuritic outgrowth by increasing VAMP expression in the differentiated PC12-Dp40 cells (Fig. 4) and decreasing it in the PC12Dp40_L170P_ cells that express the mutant Dp40_L170P_. The lack of long neurites observed in the PC12-Dp40_L170P_ cells could be associated with the nuclear distribution of the mutant Dp40_L170P_ protein (Fig. 5) by regulating VAMP expression through its decrease and preventing the Dp40 interaction with VAMP and with other synaptic vesicle proteins (SNAP25 and STX1A), disrupting the exocytosis cycle and therefore its participation in neurite outgrowth^39^. Considering the facts mentioned, it is possible that the disruption of VAMP as a consequence of the nuclear localization of Dp40_L170P_ in the differentiated PC12-Dp40_L170P_ cells promotes the α-internexin overexpression as a scaffold neurofilament to restore the reassembly of neurofilaments and therefore neurite outgrowth.

However, previous reports have shown that overexpression of Dp71_Δ78-79_ and Dp71e_Δ71_ stimulates neurite outgrowth in NGF-differentiated PC12 cells, and both cell lines presented a high expression of HspB1^17,18^. Because Myc-Dp40 overexpression promotes neuronal differentiation (Fig. 1), we tested the expression level of HspB1 in the differentiated PC12 cells. Interestingly, the expression of this protein was very low in the differentiated PC12 cells (Fig. 4). This finding indicates that Dp71 and Dp40 promote neurite outgrowth through a different pathway. Thus, our results suggest that Dp40 also plays an important role in the neuronal differentiation process through the regulation of the expression of proteins related to synaptic vesicles such as VAMP and neurofilaments such as α-internexin, possibly through its differential subcellular distribution in PC12 cells. Analysis of the mechanisms that stimulate the disruption of neuronal differentiation as a consequence of the subcellular distribution of Dp40 should be the next step. Taken together, these results suggested that the neurite outgrowth promoted by Dp40 overexpression could be carried out through a different strategy than the increase in HspB1, as was previously reported in PC12 cells that overexpress dystrophin Dp71^17,18^, possibly through VAMP expression, which is increased in the differentiated PC12-Dp40 cells but decreased in the PC12-Dp40_L170P_ cells.

In summary, in this work, we reported the effect of Dp40 and the mutant Dp40_L170P_ on the neuronal differentiation process of PC12 cells. Dp40 expression promotes an increase in the percentage of cells with neurites as well as in neurite length, while Dp40_L170P_ expression decreases neurite length. Dp40_L170P_ modifies the protein expression profile of PC12 Tet-On cells, upregulating the expression of proteins involved in the cytoskeletal reorganization and structural proteins such as α-internexin and S100a6. Additionally, during neuronal differentiation, Dp40 overexpression increased the expression of VAMP, in contrast to Dp40_L170P_, which decreased it. However, the low expression of HspB1 in differentiated PC12-Dp40 cells suggests that Dp40 promotes neurite outgrowth through a different pathway than of dystrophin Dp71e_Δ71_ and Dp71_Δ78-79_. These data support the hypothesis that the disruption of Dp40 alters neurite outgrowth and could contribute to the cognitive deficit present in DMD patients with mutations in residue 170 of dystrophin Dp40.

## Materials and methods

### Vector construction

The cDNA fragments of Dp40 and Dp40_L170P_ were obtained from the vectors pcDNA4/HisMax-TOPO/Dp40 and pcDNA4/HisMax-TOPO/Dp40_L170P_^3^ through amplification by PCR using the primers 5′TAGATCACGCGTACATGAGGGAACACCTCAAAGGC3′ (pTRE-MluI) and 5′GATCTAGCGGCCGCTCACGTTTCCATGTTGTCCCCCTCTAAC3′ (Dp40-NotI), which add the restriction sites MluI and NotI, respectively. The DNA sequence of Dp40 corresponds to the *Rattus norvegicus* sequence reported in GenBank (KF154977.1). With T4 DNA ligase, the Dp40 and Dp40_L170P_ fragments were cloned into pGEM-T Easy (Promega, Madison, WI, USA) as a transient vector and then sequenced. After Dp40 and Dp40_L170P_ fragment excision with restriction enzymes and cloning in the vector pTRE2pur-Myc (Clontech, Mountain View, CA, USA.), which adds a Myc flag tag to the N-terminal end of Dp40 and Dp40_L170P_, an inducible expression system Tet-On was generated using the vectors pTRE2pur-Myc/Dp40 and pTRE2pur-Myc/Dp40_L170P_.

### Cell culture and NGF differentiation

PC12 Tet-On cells (PC12 cells that express a regulator protein from the Tet-On system) were purchased from Clontech laboratories (Cat. 631137). pTRE2pur-Myc/Dp40 and pTRE2pur-Myc/Dp40_L170P_ vectors were used to stably transfect PC12 Tet-On cells. Then, we generated a pool of puromycin resistant PC12-Dp40 and PC12-Dp40_L170P_ cells to isolate clones that express the recombinant proteins Myc-Dp40 and Myc-Dp40_L170P_ in an inducible manner. As a control, PC12 Tet-On cells were stably transfected with an empty pTRE2pur-Myc vector to obtain PC12 control cells as previously described^41^. All cell lines were cultured in Dulbecco’s modified Eagle’s medium (Gibco, Rockville, Maryland, USA) supplemented with 10% heat-inactivated horse serum, 5% Tet System Approved foetal bovine serum, 100 U/ml penicillin, 1 mg/ml streptomycin, 250 ng/ml mycostatin, and 100 μg/ml geneticin (G418). The expression of Myc-Dp40 and Myc-Dp40_L170P_ was induced with 100 ng/ml doxycycline for 24 h to obtain undifferentiated PC12 cells. For NGF-differentiated PC12 cells, induction was maintained throughout the differentiation process. Under differentiation conditions, the medium supplemented with 100 ng/ml doxycycline and 50 ng/ml NGF was changed every 3 days for 9 days^42^.

### Morphometric analysis of neurite outgrowth

PC12-Dp40, PC12-Dp40_L170P_ and PC12 control cells were plated at low confluence on collagen-coated plastic dishes and cultured in the presence of NGF for 9 days. Three independent experiments were performed for each cell line and ten micrographs were taken in each experiment using an inverted microscope (Axiovert. A1 Zeiss) with 10× objective. Data from the three independent experiments are as follows: for PC12 control cell line a total of 554 cells (213, 140 and 201 for each experiment) were analyzed. For PC12-Dp40 and PC12-Dp40_L170P_, a total of 1,061 cells (390, 281 and 390 for each experiment), and 431 cells (256, 88 and 87 for each experiment) were analyzed, respectively. To quantify the neurite outgrowth ratio, we considered all cells that produced a neurite greater than a cell body and the percentage of differentiated cells was obtained in each experiment and cell line as follow: number of cells with neurites/total number cells × 100. For neurite length, all cells with neurites greater than two cell bodies were considered and the average of neurite length was obtained from the three experiments for each cell line. Axiovision software (Carl Zeiss™, AxioVision Rel. 4.8.2, https://carl-zeiss-axiovision-rel.software.informer.com/4.8/) was used to count the neurites and to measure their length.

### Protein extraction and 2-DE

For PC12 control and PC12-Dp40_L170P_ cell lines, protein samples were obtained from three independent experiments and protein concentrations were determined by the Bradford method. Two-hundred µg of total protein extracts of the NGF-differentiated PC12 control and PC12-Dp40_L170P_ cells were run in 2-DE gels as previously described^17^. Isoelectric focusing and SDS-PAGE gels were run in triplicate for each cell line at the same time. The 2-DE gels obtained were stained using Bio-Safe Coomassie Stain (Bio-Rad, Hercules, CA, USA) according to the manufacturer´s instructions.

### Scanning and image analysis

The stained 2-DE gels were scanned with an Image Quant 4000 instrument (GE Healthcare, Chicago, IL, USA). For determination of the protein abundance, the percent volume (% volume) of each spot was calculated using DeCyder 2D 7.0 software (GE Healthcare, Cat. 9435-83). Spots were manually examined to eliminate artefacts. The nonparametric Mann–Whitney test was used to compare data from each group and detect spots with different expression levels. Spots with *P* values < 0.05 and changes of 1.1-fold or more were considered statistically significant. Spots of interest were excised from the 2-DE gels for identification using mass spectrometry (MS).

### In-gel trypsin digestion and protein identification by MS

The selected protein spots were excised and placed in Eppendorf tubes with 50 μl of destaining solution (50% v/v methanol and 5% v/v acetic acid), followed by washes with Milli-Q H_2_O. Gel fragments were dehydrated by incubation in 100 μl of acetonitrile (ACN) for 10 min, and the supernatant was then removed; this step was repeated once. Dry gel pieces were rehydrated with 200 ng of trypsin (Promega V528A) in 50 mM NH_4_HCO_3_ and 5% ACN and incubated overnight at 37 °C. The resulting peptides were extracted with 40 μl of 50% ACN and 5% formic acid, and the solution volume was reduced in a concentrator (Eppendorf 5301). Peptides from each sample were desalted on C18 columns (ZipTipC18). A 1:1 mixture of peptide solution and matrix solution (5 mg/ml CHCA 50% v/v and TFA 0.1% v/v) was analyzed using a 4800 Plus MALDI TOF/TOF mass spectrometer (Sciex). The search was performed with the enzyme specificity of trypsin, and one missed cleavage was allowed. The detected protein threshold was 66%, and the precursor mass tolerance was 0.5–1 Da. The MS data were compared with the *Rattus norvegicus* database (downloaded in September 2016) using Protein Pilot™ software (version 2.0.1) and the Mascot algorithm^43^.

### Western blotting

Western blotting (WB) was performed with 40–80 µg of protein extracts as previously described^42^. The mouse monoclonal antibodies anti-β-actin (1:500), anti-c-Myc (1:200) and anti-VAMP1/2 (1:200) were purchased from Santa Cruz Biotechnology (Dallas, TX, USA). Rabbit monoclonal anti-α-internexin (1:10,000), mouse monoclonal NF-L (1:500) and rabbit polyclonal HspB1 (1:500) antibodies were purchased from Abcam (Burlingame, CA, USA). The relative expression of proteins was determined by the densitometric scanning of bands using ImageJ1.3j software (US National Institutes of Health, https://imagej.nih.gov/ij/download.html) and was represented as relative density to β-actin which was used as a loading control.

### Indirect immunofluorescence microscopy

Indirect immunofluorescence (ImmF) was carried out in undifferentiated and NGF-differentiated PC12 Tet-On cells as previously described^3^. Alexa Fluor 488- and Alexa Fluor 594-conjugated secondary antibodies (Invitrogen, Life Technologies, NY, USA) were used to detect the primary antibody signal. Images were captured using a Leica confocal microscope (Leica TCS SP8) with a 40× objective at zoom 2. The fluorescence intensity ratio was quantified from equatorial Z-sections using ImageJ1.3j software (US National Institute of Health, https://imagej.nih.gov/ij/download.html). Differential subcellular distribution of c-Myc, Myc-Dp40 and Myc-Dp40_L170P_ proteins was obtained from three independent experiments. Fifteen cells were analyzed for each experiment being a total of 45 cells for each cell line. For this, the sum of immunofluorescence intensity from both nucleus and cytoplasm was considered as 100% and the percentage of immunofluorescence intensity of nucleus or cytoplasm was obtained.

### Statistical analysis

Data are shown as the mean ± SD of three independent experiments. Statistical analyses were performed using Student’s t test with GraphPad Prism 5.00.288 software (San Diego, CA). *P* values < 0.05 were considered statistically significant.

## Supplementary Information

Supplementary Information.

## Data Availability

All data are available in the paper and electronic supplementary material.
