# Supplementary material for: Overexpression of the dystrophins Dp40 and Dp40L170P modifies neurite outgrowth and the protein expression profile of PC12 cells
Source: Sci Rep. 2022 Jan 26;12:1410. doi: 10.1038/s41598-022-05271-2 (PMC8791958; doi:10.1038/s41598-022-05271-2)
Supplement: Supplementary file 1 — Supplementary Information. [file 41598_2022_5271_MOESM1_ESM.pdf]

Overexpression of the dystrophins Dp40 and Dp40$_{L170P}$ modifies neurite outgrowth and the protein expression profile of PC12 cells

César García-Cruz, Candelaria Merino-Jiménez, Jorge Aragón, Víctor Ceja, Brenda González-Assad, Juan Pablo Reyes-Grajeda & Cecilia Montanez

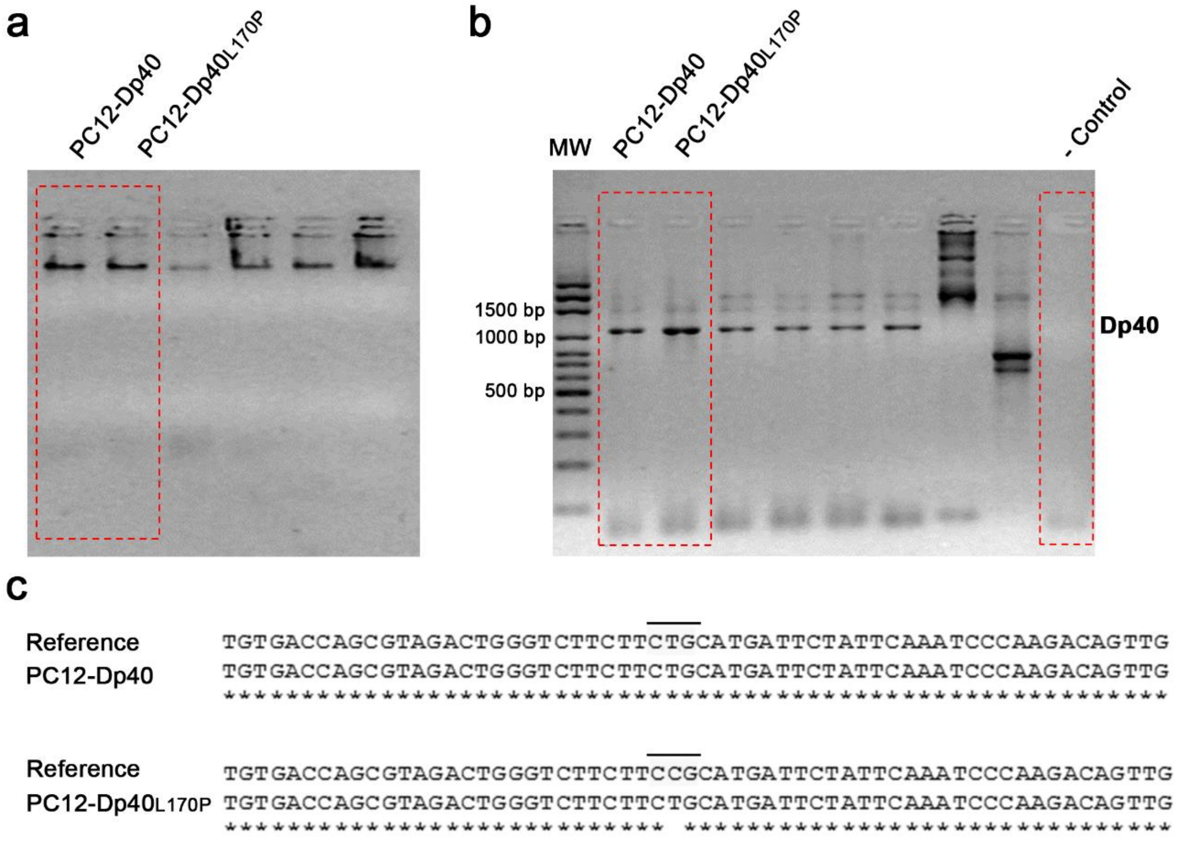

Figure S1. Insertion of pTRE2pur-Myc/Dp40 and pTRE2pur-Myc/Dp40$_{L170P}$ vectors in the genomic DNA of the PC12-Dp40 and PC12-Dp40$_{L170P}$ cells. (a) Extraction of genomic DNA through the phenol-chloroform technique was obtained from the undifferentiated PC12-Dp40 and PC12-Dp40$_{L170P}$ cells (red dotted box). (b) PCR of genomic DNA using the oligos rATGDp71 (5´ATGAGGGAACACCTCAAAGGCCACG3´) and pTRE-3´ (5´AGTTTGGGGACCCTTGATTGTT3´). The first column corresponds to the molecular weight (MW) marker in base pairs. The second and third columns correspond to the amplified fragments of Dp40 and Dp40$_{L170P}$, respectively (red dotted box). The last column corresponds to the negative control, without DNA (- Control, red dotted box). (c**)** The reported sequence of Dp40 (KF154977.1) was aligned with the sequences of Dp40 and Dp40$_{L170P}$. The codon changes between Dp40 and Dp40 $_{L170P}$ are highlighted with a line.

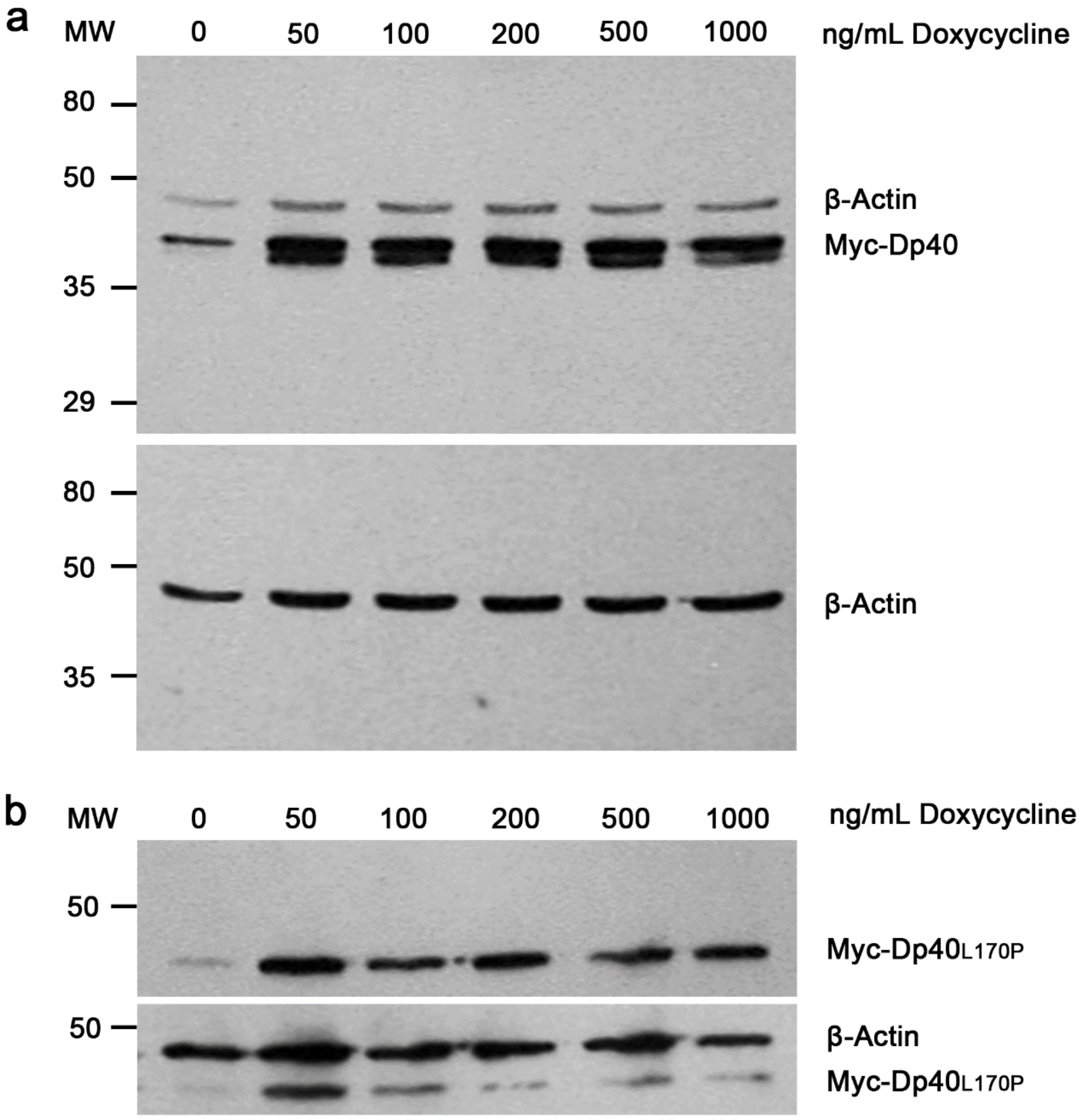

Figure S2. Induction curve of the recombinant proteins Myc-Dp40 and Myc-Dp40$_{L170P}$ treated with doxycycline. Protein extracts were obtained from the undifferentiated PC12-Dp40 (clone SC2) and PC12-Dp40$_{L170P}$ (clone C7) cells at increasing doxycycline concentrations and analyzed by WB. (a) Expression of Myc-Dp40 protein in the PC12 Dp40 cells. (b) Expression of the Myc-Dp40$_{L170P}$ protein in the PC12-Dp40$_{L170P}$ cells. A representative image of two independent experiments is shown. The absence of images of complete blots is due to the shortage and delay in material supply. Blots were cut prior to hybridization as well as to the exposition to the radiographic film. β-actin was used as a loading control. MW: molecular weight is indicated in kDa.

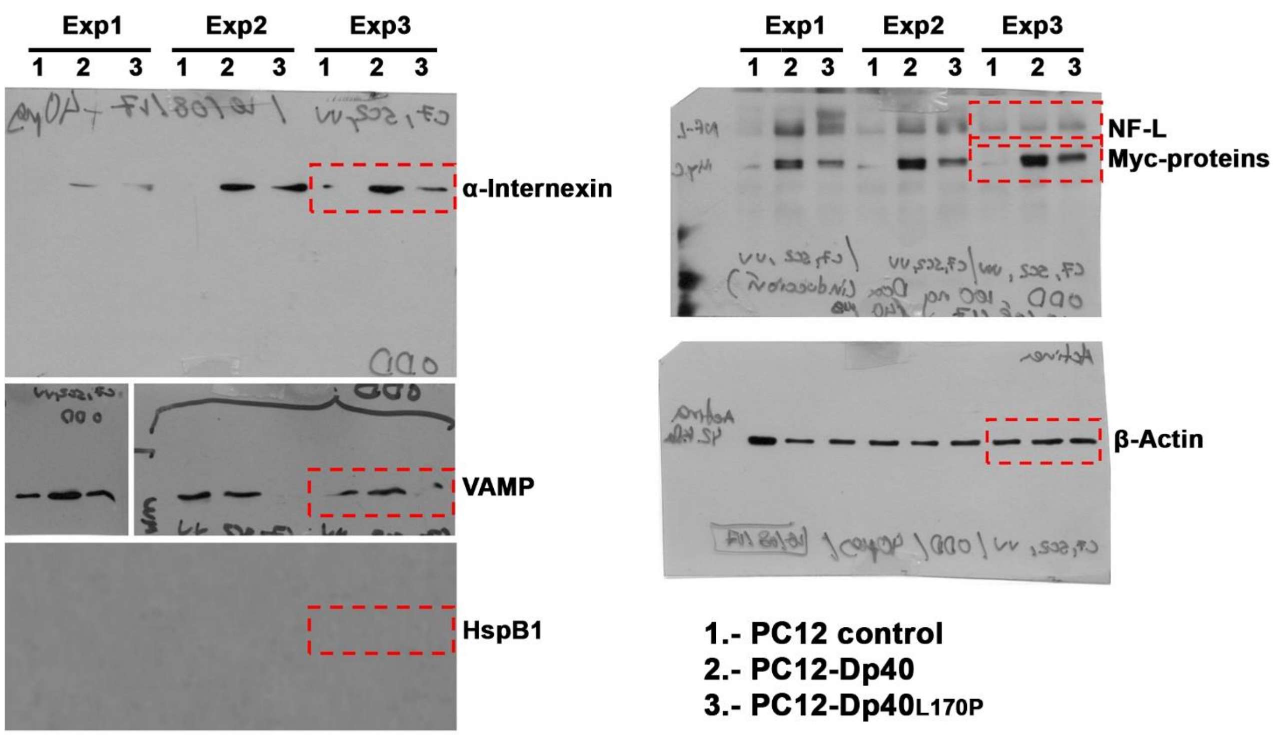

Figure S3. Original blots showing the expression of the proteins α-internexin, VAMP, HspB1, NF-L, Myc-Dp40, Myc-Dp40$_{L170P}$ and β-actin in the undifferentiated PC12-Dp40 and PC12-Dp40$_{L170P}$ cells of the three independent experiments. Red dotted box corresponds to the area cropped and showed in the figure 3a of the main paper. The absence of images of complete blots is due to the shortage and delay of material supply. All blots were cut prior to hybridization as well as to the exposition to the radiographic film.

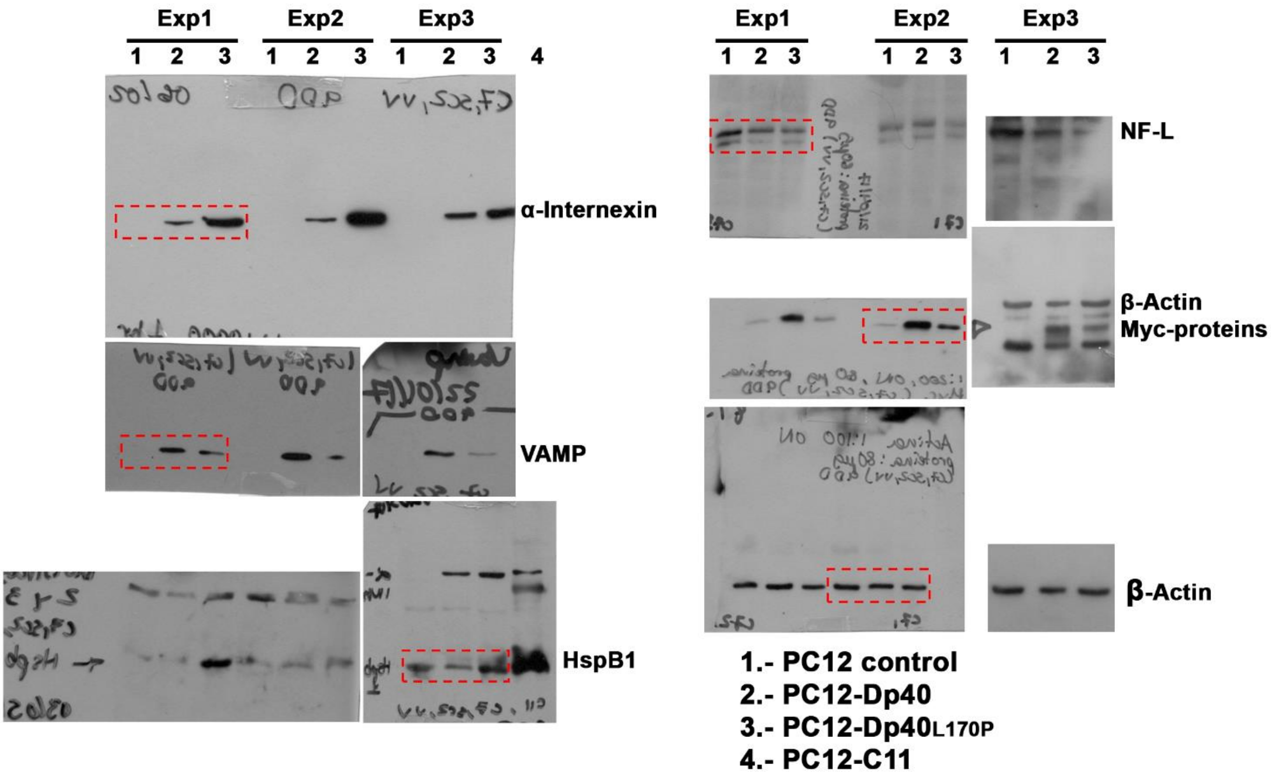

Figure S4. Original blots showing the expression of the proteins α-internexin, VAMP, HspB1, NF-L, Myc-Dp40, Myc-Dp40$_{L170P}$ and β-actin in the NGF-differentiated PC12-Dp40 and PC12-Dp40$_{L170P}$ cells of the three independent experiments. Red dotted box corresponds to the area cropped and showed in the figure 4a of the main paper. The absence of images of complete blots is due to the shortage and delay of material supply. All blots were cut prior to hybridization as well as to the exposition to the radiographic film. PC12-C11 cells were used as positive control, in which a high expression of HspB1 was observed as a consequence of stable expression of Dp71$_{\Delta 78\text{-}79}$, a dystrophin mutant that stimulates PC12 wild-type differentiation (Reference 17 in the main paper).
